# HDAC Family Members Intertwined in the Regulation of Autophagy: A Druggable Vulnerability in Aggressive Tumor Entities

**DOI:** 10.3390/cells4020135

**Published:** 2015-04-23

**Authors:** Emily Koeneke, Olaf Witt, Ina Oehme

**Affiliations:** 1 Clinical Cooperation Unit Pediatric Oncology, German Cancer Research Center, Im Neuenheimer Feld 280, 69120 Heidelberg, Germany; E-Mails: e.koeneke@dkfz.de (E.K.); o.witt@dkfz.de (O.W.); 2 Section of Pediatric Brain Tumors, Department of Pediatric Oncology, Hematology and Immunology, University Hospital Heidelberg, 69120 Heidelberg, Germany

**Keywords:** histone deacetylase inhibitor, cancer, HDAC6, HDAC10, autophagic flux, targeted therapy

## Abstract

The exploitation of autophagy by some cancer entities to support survival and dodge death has been well-described. Though its role as a constitutive process is important in normal, healthy cells, in the milieu of malignantly transformed and highly proliferative cells, autophagy is critical for escaping metabolic and genetic stressors. In recent years, the importance of histone deacetylases (HDACs) in cancer biology has been heavily investigated, and the enzyme family has been shown to play a role in autophagy, too. HDAC inhibitors (HDACi) are being integrated into cancer therapy and clinical trials are ongoing. The effect of HDACi on autophagy and, conversely, the effect of autophagy on HDACi efficacy are currently under investigation. With the development of HDACi that are able to selectively target individual HDAC isozymes, there is great potential for specific therapy that has more well-defined effects on cancer biology and also minimizes toxicity. Here, the role of autophagy in the context of cancer and the interplay of this process with HDACs will be summarized. Identification of key HDAC isozymes involved in autophagy and the ability to target specific isozymes yields the potential to cripple and ultimately eliminate malignant cells depending on autophagy as a survival mechanism.

## 1. Introduction

In eukaryotic systems, multiple forms of autophagy have evolved to maintain homeostasis, and these processes interact with one another as well as cell death pathways to determine the cell’s fate [1,2]. Autophagic activity can be a non-specific and bulk-degradative process, but many selective forms of autophagy have also been described [3,4,5]. A rapidly growing body of literature is elucidating the role of these pathways in cellular physiology and fate [4,5]. Most of the time, the term “autophagy” refers to one type of autophagy, macroautophagy, and this will also be the case throughout this review. In healthy cells, basal autophagy serves to recycle proteins or organelles that are either non-functional or dysfunctional or simply no longer necessary. Intracellular signaling as well as signaling from the microenvironment can either stimulate or restrict this process, depending on the energy needs of the cell or whether stressors have been encountered [1,2,6]. Dysfunctional autophagy has been associated with several disease states, which include cancer, neurodegenerative diseases, inflammatory conditions, and immune disorders [1,2,7]. Strict regulation of these pathways is important, since aberrant autophagy, while not inevitably fatal to the cell, alters its physiology significantly and can promote or maintain a dysfunctional state, such as the unrestricted growth seen in malignancy.

The histone deacetylase (HDAC) family is comprised of 11 enzymes, which are grouped into four separate classes based on their homology to yeast proteins. Members of this evolutionarily-conserved enzyme family are involved in pivotal cellular processes such as proliferation, differentiation, and autophagy, with established roles in both cytoplasmic signaling as well as epigenetic regulation of gene expression via participation in transcription complexes and histone acetylation [8]. A fundamental feature of cancer in general is the deregulation of vital cell signaling pathways and genetic alterations that result in aberrantly high or silenced expression of proteins involved in any process that would promote or restrict their uncontrolled proliferation, respectively [9]. Thus, it is no surprise that aberrant expression of members of the HDAC family has been associated with a variety of malignancies, including lung cancer, breast cancer, colon cancer, neuroblastoma, medulloblastoma, and pancreatic carcinoma, to name a few [10,11,12,13,14,15]. The role of these enzymes in tumorigenesis is broad, ranging from changing gene expression via histone deacetylation to deacetylation of cytoplasmic proteins, resulting in signaling changes and disrupting essential processes, such as autophagy [10,11,15]. As a result of this relationship to tumorigenesis, small molecule inhibitors of HDACs (HDACi) have been of great interest to the medical community for the treatment of cancer. Two broad-spectrum HDACi, vorinostat and romidepsin (FK228), have already been approved for advanced cutaneous T-cell lymphoma in the United States and Australia, and numerous clinical trials are under way to investigate vorinostat and other broad-spectrum HDACi in both solid and hematologic malignancies [16,17,18,19,20,21]. With the knowledge that individual HDAC isozymes have unique roles, more selective inhibitors have been developed, which target only one or two isozymes [22].

In this review, the involvement of individual HDAC family members in the regulation of autophagy will be discussed in the context of aggressive tumor entities. With the development of novel small molecule inhibitors that target specific HDAC isozymes, an opportunity emerges to cripple cancer cells by interfering with autophagy in a manner that exploits the vulnerability created by an exaggerated reliance upon this process.

## 2. The Role of Autophagy in Cancer

In recent years, the role of autophagy in the development, maintenance, and progression of cancer has been extensively explored [1,23,24]. Autophagy is not only important for intracellular dynamics, it has also been shown to modulate the cell’s interaction with immune cells and the surrounding microenvironment [25,26,27].

As with many intracellular processes, context is important for determining the outcome of a given alteration. The functional consequence of autophagy on tumor development and progression is dependent on cell type, tumor suppressor and/or oncogene mutation status. The mechanisms by which autophagy can be both tumor promoting and suppressive are complex [23,24,28]. While some studies have shown a tumor suppressive function of autophagy by knocking out genes important for autophagy induction [29,30,31,32,33], others have shown that the knockdown of autophagy related genes impairs autophagic flux and promotes cancer cell death in metabolically unfavorable conditions as well as in response to cytotoxic chemotherapy [34,35,36].

### 2.1. Targeted Blockade of Autophagic Flux as a Therapeutic Intervention

Some anticancer treatments, such as the cytotoxic drugs doxorubicin and cisplatin and the alkylating agent temozolomide as well as ionizing irradiation, have been associated with induction of autophagy [37,38,39,40]. Blocking autophagic flux in cancer cells *in vitro* resulted in very promising sensitization to anticancer treatment [11,40,41,42,43,44,45]. Hence, clinical trials have been initiated using regimens that combine conventional chemotherapy or other agents with autophagic flux-blocking agents, such as chloroquine, in an attempt to sensitize the tumors to therapy [39,46]. Chloroquine (CQ) and its hydroxylated derivative, hydroxychloroquine (HCQ), are lysosomotropic agents and inhibit lysosomal functions through concentration in acidic vesicles and therefore block autophagic flux at the level of degradation [47,48]. However, CQ and HCQ have properties that are not limited to acidification. Their accumulation in lysosomes has been also linked to lipase inhibition and lysosomal destabilization, and they have also been shown to weakly intercalate with DNA, causing DNA damage, and, finally, CQ has been shown to induce p53 and p21^WAF^ and cause cell cycle arrest [49]. Though they are effective autophagosome degradation inhibitors, these agents additionally affect a diversity of other cellular processes, which should be kept in mind when evaluating clinical trial results and reported treatment side effects.

Most of the early clinical trials initiated for the combination of HCQ with anticancer therapy were dose-finding in nature and were not primarily designed to address clinical efficacy. However, in a study combining temozolomide and HCQ, evidence for impaired autophagic flux in peripheral monocytes and in several patients, stable disease or a partial response was achieved [39]. In one patient with advanced melanoma, a durable response of greater than one year was seen [39]. Also, a trial examining the effects of HCQ in combination with temozolomide and radiation therapy in glioblastoma found that HCQ treatment was able to block autophagic flux in peripheral blood mononuclear cells (PBMCs) [46]. However, the maximum tolerated dose of HCQ was rather low and no significant improvement in overall survival was observed with added HCQ [46].

In all of these studies, high grade toxicities were identified in patients receiving HCQ at the dose associated with the best outcomes plus chemotherapy [39,46]. The most common toxicities seen with combination treatment at all dose levels of HCQ, but with greater frequency at the highest dose levels, were anorexia and nausea. Other common toxicities that were observed, but were less severe, were fatigue, rash, stomatitis, lymphopenia, thrombocytopenia, diarrhea, dizziness, and constipation. The increased hematologic toxicities seen with continuous dosing in one study suggest that intermittent compared with continuous dosing may allow for dose escalation [46,50]. Thus new, less toxic and more specific autophagic flux inhibiting compounds, which create a larger therapeutic window are needed. In addition, identifying which patients would be most likely to benefit from therapy combining autophagy-inhibiting agents remains a challenge. The relationship between the effects of autophagy-modulating drugs in the context of a human tumor compared with cell culture and animal models is complex and not directly translatable [50]. One common method to identify candidates for targeted therapy is by gene mutation status. Indeed, oncogene and tumor suppressor gene status also affect the interplay between autophagy and tumorigenesis as well as tumor progression [51,52]. For example, *KRAS* mutations and constitutive autophagy upregulation are closely connected. Differential effects of autophagy inhibition have been observed in *RAS*-mutated and non-mutated cancer cells [34,35], and it has been observed that p53 status can also modulate this effect [53]. It is worth noting that there is also evidence that WT *RAS* can stimulate autophagy activation under conditions of stress [54], thus examining levels of basal autophagy instead of mutation status may be warranted.

### 2.2. Pitfalls of Using Autophagic Flux Inhibitors as Adjunct Therapy to Anticancer Treatment

Several factors hamper a clear interpretation of the outcomes of clinical trials investigating autophagic flux modulation as a part of anticancer treatment. Many studies investigate autophagic flux in PBMCs as a surrogate marker of on-target activity of autophagy inhibitors. However, autophagic flux changes in PBMCs do not always reflect the degree to which autophagy is affected in the tumor itself [50]. Further complicating the matter is that the observation of increased autophagic vacuoles in tumor samples does not allow one to distinguish between autophagy induction and inhibition [50], necessitating measurement of pre- and post-treatment biomarkers to assist in monitoring and interpreting treatment response. Thus, a reliable biomarker to identify autophagic flux in tumors, allowing assessment at baseline and during treatment, remains elusive [50]. Another aspect that has to be taken into account is that it is very likely that different cancer entities have different basal levels of autophagic flux. Blocking autophagic flux in cells that either have a high basal level or an induction secondary to stress from chemotherapy or radiation has been shown to be associated with a high degree of reactive oxygen species (ROS) accumulation [55,56]. In a study examining acute megakaryocytic leukemia, the basal level of autophagy was reported to be very low, and this in itself was a vulnerability to autophagic flux inhibition [57]. These factors will need to be explored in different malignancies to identify the level of basal autophagy and how that relates to that entity’s susceptibility to autophagic flux inhibition.

To further complicate this issue, selective autophagy, such as mitophagy, might play a very important role in mediating treatment resistance mechanisms. Normally mitochondria undergo specific degradation via mitophagy, a pathway which ultimately leads to delivery of the defective mitochondria to autophagosomes [3,58,59,60]. Blocked mitophagy leads to an accumulation of defective mitochondria along with ROS and cell death [58].

Easily overlooked in cell culture studies or experiments with immune-deficient mouse models is the interplay between autophagy and immune response. Cells undergoing autophagy secrete factors, such as ATP, into the extracellular space, very likely via lysosomal exocytosis. ATP secretion is necessary to recruit immune cells, such as T lymphocytes and dendritic cells, to induce an immunogenic cell death of the cancer cells [61,62,63]. When murine colon adenocarcinoma cells were defective for autophagy induction via ATG5 and BECN1 depletion, they were less sensitive to radiotherapy than in those with intact autophagy when implanted in an immunocompetent host [64]. Similarly, autophagy-deficient cancers due to ATG5 and ATG7 depletion failed to attract immune cells to the tumor and thus had a less robust response than autophagy-competent tumors [61]. On the other hand, cells with dysfunctional autophagy due to a conditional knockout of the autophagy regulator, FIP200, seem to be more sensitive to environmental stimuli and release more chemokines and interferon, recruiting immune response and suppressing tumor growth and development [52]. In any case, it is important to consider the host immune response and the interaction of active or dysfunctional autophagy with immune effector cells.

## 3. The Histone Deacetylase Family and Its Role in Cancer

A diverse group of lysine deacetylating enzymes, the histone deacetylase (HDAC) family is divided into four subgroups based on phylogenetic analysis, which followed the initial discovery of sequence similarity between the human HDAC1 and the yeast Rpd3, which had already been identified as an important gene regulator (reviewed in [8,65]). The name of this family belies the complexity of the roles these enzymes play in cell physiology. Not only do they deacetylate histones, different members have been found to deacetylate many other non-histone proteins, both in the nucleus and in the cytoplasm, and many proteins throughout the cell are post-translationally acetylated, yielding a broad selection of targets for histone deacetylases [66]. Classes I, II and IV comprise the “classical” HDACs, which are Zn^2+^-dependent enzymes, in contrast to the Class III sirtuins, whose seven members are NAD^+^-dependent enzymes. In this chapter, we will give an overview of the “classical” HDAC family and their importance in cancer. For more detailed information we also refer to ([10,22,67,68], and references therein).

### 3.1. Class I HDACs and Their Role in Cancer

Class I HDACs share homology with the yeast Rpd3. This class includes HDACs 1, 2, 3, and 8, and its members are expressed ubiquitously in humans. Members 1, 2, and 3 are found in multi-protein repressor complexes in the nucleus, where they regulate gene expression [8,69]. HDAC8 is found in both the nucleus and cytoplasm, and while it has not been found in any multi-protein complexes, it has been described to be involved in transcriptional repression [70]. Since its initial characterization, some deacetylation targets have been identified and these include the tumor suppressor, ARID1A, the retinoic acid induced gene RAI1 and the transcriptional regulator NCOA3 [71], as well as a protein important for sister chromatid cohesion, structural maintenance of chromosomes 3 (SMC3) [72].

Knockout mouse studies revealed important functions for class I family members, since the deletion of each class I HDAC was lethal [73]. Even the close relatives Hdac1 and Hdac2 were not completely redundant in function in the developing mice. Knockout of Hdac1 is embryonic lethal (E10.5) due to cell proliferation defects. Deletion of Hdac2 leads to perinatal mortality (P1) secondary to defects in cardiac morphology [74]. Hdac3-null mice die before embryonic day 9.5 due to cell cycle defects and DNA damage due to impaired double-strand break repair [75]. Hdac8 controls patterning of the skull by repressing a specific subset of transcription factors in cranial neural crest cells. Deletion of Hdac8 in mice leads to perinatal (P1) lethality due to skull instability [76].

High expression levels of class I HDACs have been reported for many different tumor entities. For example, high expression of HDACs 1, 2, and 3 are associated with poor outcomes in colorectal, prostate and gastric cancers, and high expression of HDAC8 is associated with advanced disease and poor outcomes in the childhood cancer, neuroblastoma [67,77]. As with autophagy, HDAC expression and activity can also play a dual role with regard to cancer initiation and maintenance. For instance, it has been reported that HDAC1 acts as an oncosuppressor in tumorigenesis of acute promyelocytic leukemia, but acts as an oncogene with regard to maintenance and progression [78].

### 3.2. Class II HDACs and Their Role in Cancer

Class II HDACs share homology with yeast Hda1, and are sub-classified into Classes IIa and IIb [8]. The former group contains HDACs 4, 5, 7, and 9, and HDACs 6 and 10 belong to group IIb.

#### 3.2.1. Class IIa HDACs

Class IIa HDACs shuttle between the nucleus and cytoplasm. This group of HDACs regulate the activity of transcription factors, such as myocyte enhancing factor-2 (MEF2), and change localization based on phosphorylation status, which is modulated by signaling pathways such as salt-inducible kinases, checkpoint kinase-1, and calcium/calmodulin-dependent kinases [8]. Unlike Class I HDACs, these family members are not ubiquitously expressed, rather exhibiting tissue-specific expression. Although the members of this Class of HDACs share a mechanism of action in their interaction with MEF2, the effects are tissue-type specific. For instance, HDAC4 has been implicated in regulation of transcription control of ossification. In its absence, extra-skeletal ossification occurs and mice lacking Hdac4 die within the first week of life as a result [79]. The Hdac5 and Hdac9 single knockout mouse models are viable. However, double knockout of Hdacs 5 and 9 leads to a high probability of the development of lethal cardiac defects [80]. These two family members are important for regulating the program important for cardiac formation in the embryo. Finally, HDAC7 is important for vascular integrity and is expressed in the vascular endothelium in early embryogenesis [81]. Homozygous deletion of Hdac7 in mouse embryonic stem cells is lethal due to loss of adhesion between endothelial cells, which leads to vascular dilatation, rupture, and hemorrhaging [81].

The expression patterns in tumors are very diverse for class IIa members. In gastric cancer, HDAC4 expression was elevated in tumor samples and upregulation was associated with increased proliferation and suppression of ROS formation [82]. However, HDAC4 dysfunction and downregulation have been also reported to be associated with cancer development [83]. HDACs 5 and 7 exhibit higher expression in colorectal cancer compared with renal, bladder and breast cancers [67]. High expression of both HDACs 5 and 9 was associated with prognostically poor subgroups in medulloblastoma [12]. HDAC5 is also aberrantly expressed in hepatocellular carcinoma together with HDAC3 [83]. High levels of cytoplasmic HDAC7 have been reported in pancreatic cancer patients and, in children with ALL, overexpression of HDAC7 and HDAC9 correlated with poor prognosis. However, HDAC9 has also been described to be downregulated in glioblastoma [83]. These data, together with the results obtained from cell culture studies, imply that dependent on the cellular context, class IIa HDACs can act as oncogenes or tumor suppressors [83].

#### 3.2.2. Class IIb HDACs

Class IIb HDACs seem to have primarily cytoplasmic roles, with HDAC6 deacetylatingalpha-tubulin and functioning as part of protein aggresome formation and processing [84,85,86]. In addition, HDAC6 has been shown to deacetylate HSP90 and plays an important role in stress response [87,88,89]. Additionally, HDAC6 has been shown to regulate acetylation of cytoplasmic Ku70, which is in a complex with Bax, which, when acetylated, promotes apoptosis via Bax release [90]. HDAC6 also deacetylates cortactin, which impacts binding to F-actin and cell motility [91]. HDAC6 is found primarily in the heart, liver, kidney, and pancreas [67]. Found in both the nucleus and the cytoplasm, HDAC10 is reported to function as a transcriptional repressor [92,93,94,95]. The ability of HDAC10 to repress transcription has been described as both deacetylase dependent [93] and independent [92]. Recently, it was reported that HDACs 9 and 10 are important for homologous recombination, and specific knockdown of either family member led to impairment of this double-strand break repair process [96]. In addition, HDAC10 has been found in a complex with the transcription factor PAX3 and KAP1 and HSC70, which is important for melanogenesis [97].

While the embryonic knockout phenotype of Hdac6, characterized by increased acetylation of alpha-tubulin and Hsp90, is not lethal, murine fibroblasts lacking Hdac6 fail to recover from oxidative stress [67]. To date, no Hdac10 knockout models have been published.

High expression of HDAC6 has been associated with tumorigenesis. High expression of HDAC6 in oral squamous cell cancer is associated with advanced stage disease. However, in breast cancer HDAC6 expression correlates positively to response to endocrine treatment and is inversely related to poor survival and large tumors [67]. In gastric cancer cells, HDAC10 was found to regulate the expression of thioredoxin interaction protein (TXNIP), indicating an importance in response to oxidative stress, and knockdown of HDAC10 resulted in accumulation of ROS and cell death [98]. In a population-based study examining HDAC10 polymorphisms it was found that one variant that resulted in increased expression of HDAC10 protein also corresponded with an increased occurrence and accelerated onset of hepatocellular carcinoma in patients with chronic hepatitis B infection [99]. In childhood tumors of the nervous system (neuroblastoma and medulloblastoma), elevated HDAC10 expression is associated with poor outcome of treated patients [11]. In a study examining HDAC isozyme expression in B-cell chronic lymphocytic leukemia, higher expression of HDAC10 and lower levels of HDAC6 were associated with a poor prognosis [100]. In contrast, in cervical cancer, elevated HDAC10 expression was associated with suppression of metastasis and downregulation of metalloproteases 2 and 9 [101]. As with HDAC6, HDAC10 has diverse roles, dependent on tissue type and context.

### 3.3. Class IV HDAC11 and Its Role in Cancer

Class IV has a single member, HDAC11, whose structure bears some resemblance to Classes I and II HDACs, but is not similar enough to be placed in either class [8,102,103]. The specific roles of HDAC11 are still being elucidated, but it is notable that its structure is evolutionarily conserved, not only in vertebrates and invertebrates, but also in plants [8]. In the developing mouse brain, it has been found that Hdac11 is expressed postnatally in a distinctive pattern, primarily in oligodendrocytes and some neurons, whereas it is hardly expressed in astrocytes [104]. In oligodendrocytes, the Hdac11 seems to play a role in cell maturation, and its absence results in fewer cell processes and decreased expression of major genes important for oligodendrocyte function [105]. In non-transformed fibroblasts, mRNA levels of HDAC11 correlate inversely with proliferative status where HDAC11 mRNA accumulates in cell cycle arrest and overexpression of HDAC11 is also growth suppressive [106].

High expression of HDAC11 has been found in several different solid tumors, including breast carcinoma, hepatocellular carcinoma, and renal carcinoma [67]. Correspondingly, depletion of HDAC11 in cell lines derived from colorectal, prostate, ovarian, and breast cancers resulted in cell death and reduction in metabolic activity, but when HDAC11 is depleted in normal cells, no effects on survival or metabolic activity are seen [107]. It has also been reported that HDAC11 may play a tumor suppressive role in pancreatic endocrine tumors [108]. In a study examining immune cells, it was found that HDAC11 is expressed differentially in myeloid cell populations and may play a role in expansion of the myeloid-derived suppressor cell population and it was also shown to be involved in down-regulating the expression of immune-suppressive cytokine, IL-10 in these cells [109]. In a murine Hdac11 knockout model, it was shown that tumor growth was more rapid in Hdac11-deficient mice compared with the wild-type mice [109].

In general, high expression of HDAC family members is found in malignancy. The aberrant expression is clearly tissue and malignancy specific, indicating that the role of these enzymes varies depending on the tissue and environment.

### 3.4. HDAC Inhibitors as Promising Anticancer Agents

After the serendipitous discovery that dimethyl sulfoxide induced differentiation in murine erythroleukemia cells, a number of compounds were synthesized to induce similar effects [110,111,112]. Among those compounds was suberoylanilide hydroxamic acid (SAHA, also known as vorinostat), which was subsequently discovered to inhibit histone deacetylases [112,113]. Since that time not only has there been much research investigating the role of HDACs in cancer physiology, but many compounds have also been developed or discovered to have inhibitory activity against histone deacetylases. Multiple compounds stemming from different structural families are able to inhibit the activity of several HDAC family members simultaneously. Some drugs, such as valproate and butyrate, with other established mechanisms of action, were later discovered to inhibit the activity of histone deacetylases. The two largest classes of histone deacetylase inhibitors are benzamides and hydroxamic acids. Examples of broad-spectrum inhibitors, which inhibit family members in multiple classes, include vorinostat, trichostatin A, and panobinostat [114,115]. A chemical phylogenetic analysis, which provided insights into structure-function relationships between HDACs and HDACi, revealed that broad-spectrum inhibitors display some selectivity, as Class IIa isozymes are poorly inhibited [116]. Other HDAC inhibitors that are class-specific but not isozyme specific include valproic acid and entinostat, class I inhibitors, and bufexamac and tubastatin A, which are class IIb inhibitors [11,117]. Lack of isozyme specificity is problematic in that many processes are targeted at once. This leads to a greater potential for undesired effects. Specific targeting of isozymes yields the potential for more efficient therapy. Not surprisingly, many substances are under development that target one or two specific members of the HDAC family, such as the recently described dual HDAC6/8 inhibitors [118].

Specific inhibitors for HDAC8 are in preclinical development and are being tested for anti-cancer efficacy [119,120]. Inhibition of HDAC6 in combination with proteasome inhibition results in synergistic toxicity to multiple myeloma cells due to accumulation of ubiquitinated proteins [121]. These findings have been confirmed using other novel HDAC6 inhibitors [122,123], one of which is now being explored in clinical trials as a single agent and in combination with other therapies for multiple myeloma [16,122].

## 4. HDACs and Their Role in Autophagy

Just as the role of autophagy in cancer is complex, so too is the modulation of autophagy by different members of the zinc-dependent HDAC family. Those members involved in gene regulation via histone deacetylation have been shown to have a role in regulating the transcription of genes essential for autophagy [124]. Additionally, HDACs involved in de-acetylation of cytoplasmic proteins, such as HDAC6 and HDAC10, have been implicated in more direct roles in the process of autophagy by regulating key players. Due to the rapid evolution of the study of autophagy and an increased understanding of the intricacy of the process, the number of readouts and the complexity thereof, have increased significantly. Methods used for autophagy detection in the cited literature of this review are included in Table 1. The discrimination between elevated and blocked autophagic flux is not so simple and should ideally be addressed with a number of experiments.

The most common methods include an assessment of changes in components of autophagic machinery (e.g., LC3-II) or substrates that are degraded by autophagy (e.g., p62). Tracking changes in LC3-II, which is specifically found bound to autophagosome and autolysosome membranes, provides an indication of whether autophagic flux is affected by a substance or genetic modification. When LC3-II levels are monitored in the presence and absence of an autophagy inhibitor (e.g., bafilomycin A (late phase) or 3-methyladenine (early phase)), the determination can be made whether the effect seen is inhibition. For instance, if inhibiting the late phase of autophagy results in a substantial increase of LC3-II compared with the treatment alone and the inhibitor alone, then it can be concluded that the treatment induces autophagy. Another indication of autophagic flux is SQSTM1/p62, a protein that links ubiquitinated proteins to LC3 in the autophagosome and is itself subsequently degraded by the process. Thus, its relative depletion serves as an indicator that autophagic flux is increased and its accumulation would suggest that it may be impaired. However, p62 is not exclusively a substrate for autophagy and steady state levels may also be affected by transcriptional regulation [125]. Altogether, this reinforces the importance of employing a range of experiments to demonstrate changes in autophagic flux. Another technique is the utilization of cells transfected with mCherry-EGFP-LC3, which allows visualization of whether autophagosomes are able to efficiently fuse with lysosomes [125]. When efficient fusion is blocked, an accumulation of yellow puncta representing autophagosomes results, due to the overlapping green and red signals of LC3 in the membrane. Efficient fusion and creation of autolysosomes would result in red puncta, due to the quenching of the green signal in the acidic environment. A selection of literature addressing the role of HDAC family members via specific knockdown or knockout in autophagy is summarized in Table 2.

HDAC family members are important on several levels for the modulation of autophagy. Depleting cells of class I HDACs induced autophagic flux, which was evidenced by accumulation of the autophagosomal marker LC3-II [126,127] and increased expression of autophagy-relevant proteins, such as Beclin1 and ATG3, both of which are important for the autophagy induction steps nucleation and vesicle elongation [127]. Conversely, the simultaneous deletion of both Hdac1 and Hdac2 in mice blocked autophagosome induction and formation [128]. Though these results do not allow a generalization to be made about the precise role of HDACs 1 and 2 in autophagy, they do indicate that it is important to consider context when interpreting the results. Based on the evidence to date, it appears that whether the cell is malignantly transformed or normal, modulates how the cell is affected by changes in enzymes important for autophagic flux.

**Figure 1:**
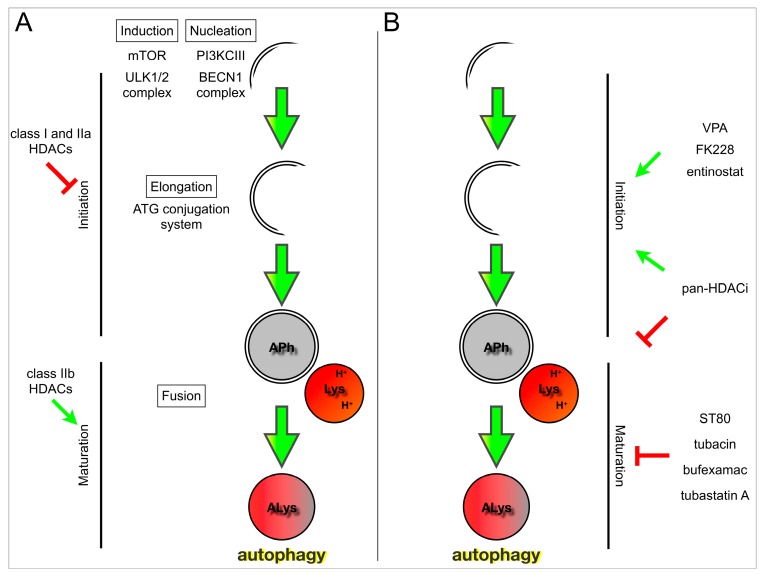
Histone deacetylase family members and their inhibitors modulate different phases of the autophagy cascade.

The knockdown of class IIa HDAC4 led to autophagy induction, with increased levels of LC3-II as well as elevated Beclin-1 and ATG7 [82]. Downregulation of HDACs 4 and 5 using miRNA-9* increased total LC3B and Rab7 (marker for late endosomes) expression levels [129]. When HDAC5 was depleted in breast cancer cells, LC3-II increased over time, and this effect was enhanced by the use of a lysosomal inhibitor, indicating that HDAC5 downregulation increased autophagic flux [130]. Thus in most studies, depletion of class I and IIa HDAC isozymes is associated with enhanced expression of autophagy regulators involved in the induction steps (Figure 1A). It is very likely that these HDACs repress important autophagy regulators and that repression is released upon HDAC depletion.

In contrast, in experiments knocking down class IIb family members, depletion of the isozymes is more associated with a block of autophagic flux (Figure 1A).

In serum-starved cervical carcinoma cells, it was found that increased LC3-II acetylation correlates with decreased autophagic flux, and that HDAC6 is at least partially responsible for deacetylating LC3-II [131]. Thus, HDAC6 depletion impaired serum starvation-induced autophagy [131]. In mouse embryonic fibroblasts, HDAC6 appears to be less important for starvation-induced autophagy but is vital for quality control (QC) autophagy [132]. In the latter form of autophagy, knockout of HDAC6 impaired fusion of autophagosomes and autolysosomes due to interference with F-actin assembly mediated by acetylation of cortactin, which was found to be specifically important for QC autophagy during which ubiquitylated proteins and aggregates are removed [132]. HDAC6 deacetylase activity was also important for the fusion of autophagosomes and lysosomes in another non-transformed model, using HEK293T cells. Here, HDAC6 controlled acetylation of salt-inducible kinase 2, a member of the AMP-activated protein kinase family [133]. In addition, HDAC6 depletion-induced block of autophagic flux sensitized breast cancer cells to treatment with the proteasome inhibitor, bortezomib, via decreased autophagic flux [134].

**Table 1:** Methods for the detection of autophagy. EM—electron microscopy; FM—fluorescent microscopy; WB—Western blot; FACS—fluorescence-activated cell sorting; PCR—quantitative real-time polymerase chain reaction; IP—immunoprecipitation; quant.—quantified.

	Methods for the Detection of Autophagy	
Method	Description	Technique
	**Morphology**	
	**Autophagosome visualization**	
autophagic vesicles ↑	increase in autophagy-associated structures	EM
LC3-GFP ↑	increase in LC3-containing autophagosomes: characteristic puncta formation	FM
LC3-ubiquitin overlapping puncta ↑	as with LC3 puncta; specific for ubiquitin-tagged proteins targeted for destruction via autophagy	FM
	**Acidic compartment dyes (autolysosomes, lysosomes)**	
AO ↑/LTR ↑/MDC puncta ↑	**A**cridine **O**range/**L**yso**T**racker**R**ed/**M**ono**D**ansyl**C**adaverine; increase in acidic compartment	FM
	**Components targeted for autophagic degradation**	
protein aggregates ↑	accelerated aggregate formation or impairment of processing	FM
mitochondria ↑	decrease in mitochondrial turnover (e.g., Tom20)	FM/WB
	**Quantification**	
	**Early-stage autophagy**	
BECN1 ↑; Vps34 ↑	accumulation or upregulation of proteins involved in early autophagy (nucleation)	WB/PCR
ATG3 ↑; ATG5 ↑; ATG7 ↑	accumulation or upregulation of protein involved in early autophagy (elongation)	WB/PCR
ac-ATG7 ↑	associated with inhibition of early autophagy	IP-WB
	**Autophagosomes and lysosomes**	
autophagosomes (EM) ↑	enriched number of autophagosomes per square millimeter	EM
LC3-II ↑	increase in LC3-conjugated autophagosomes	WB
LC3-GFP ↑; LC3-GFP ↓	increase in autophagosomes; decrease after fusion with acidic compartment	FACS
ac-LC3-II ↑	associated with decreased autophagy	IP-WB
LC3B ↑; GABARAP ↑	ATG8 homologues; transcriptional upregulation or increase in autophagosomes	WB/PCR
RAB7 ↑	accumulation or upregulation of protein involved in late autophagy (fusion); marker for endosomes	WB/PCR
LAMP2 ↑	lysosome-associated membrane protein 2; increase in lysosomes	WB
	**Acidic compartment dyes (autolysosomes, lysosomes)**	
AO ↑; LTR ↑; MDC puncta ↑	**A**cridine **O**range/**L**yso**T**racker**R**ed/**M**ono**D**ansyl**C**adaverine; increase in acidic compartment	FACS
Cyto-ID ↑	cationic amphiphilic tracer; increase in autophagic structures	FACS
	**Flux studies**	
+ early autophagy inhibitor	e.g., 3-methyladenine: early stage autophagy inhibitor; should decrease autophagosomal markers	WB (LC3-II, ATG7)/ FM-quant. (EGFP-LC3, MDC)/
+ late autophagy inhibitor	late autophagy inhibitors: NH4Cl, CQ, bafilomycin; should increase autophagosomal markers	WB (LC3-II)/ FACS (EGFP-LC3)
+ lysosomal protease inhibition	e.g., Pepstatin A/E64d; should increase autophagosomal markers	WB
p62/SQSTM1 ↑	accumulation, marker for inhibition of late stages of autophagy	WB
p62 ↓ and + late stage autophagy inhibitor p62 ↑	increase autophagic flux	WB
tandem fluorescent-tagged LC3	mCherry-EGFP-LC3B or mRFP-GFP-LC3B: Yellow puncta reflect colocalized red and green signals, representing autophagosomes; red puncta represent successful fusion to autolysosomes	FM-quant.
	**Indirect flux measurements**	
p-p70S6K ↓	indicates block of mTOR pathway	WB
p-mTOR ↑	activation of mTOR pathway, leading to an inhibition of autophagy	WB
p-AMPK ↑	activation of AMPK signaling which inhibits mTOR	WB
+ mitophagy inducer	e.g., Parkin-dependent mitophagy (Tom20 degradation/accumulation)	WB

**Table 2:** Summary of classical HDAC family members and their effect on autophagy. KD—knock-down; KO—knock-out; HCC—hepatocellular carcinoma; EM—electron microscopy; 3-MA—3-methyladenine; MM—malignant melanoma; n.d.—not determined; FM—fluorescent microscopy; GC—gastric cancer; WM—Waldenströms macroglobulinemia; MEF—mouse embryonic fibroblasts; MG132—proteasome inhibitor; QC—quality control; BORT—bortezomib; Smoke—cigarette smoke (shown by authors to induce autophagy); mitochond. dysfunction—mitochondrial dysfunction induced by CCCP—carbonyl cyanide m-chlorophenylhydrazone, a mitochondrial uncoupler; NB—neuroblastoma.

Class	Member	Targeted by	Context	Stress Status	Methods for the Detection of Autophagy			Overall Effect of HDAC KD/KO on Autophagy	Citation
					Morphology	Quantification	Flux Studies		
**I**	HDAC1	siRNA	HCC	nutrient rich	autophagic vesicles (EM) ↑	LC3-II ↑	−3-MA: LC3-II ↑ +3-MA: LC3-II ↓	Induced. 72 h post-transfection	[126]
	HDAC1	shRNA	MM	nutrient rich +p73	n.d.	BECN1 ↑ ATG3 ↑ LC3-II ↑	n.d.	Induced. 48 h post-transfection	[127]
	Hdac1 and Hdac2	double knockout	mouse (skeletal muscle)	nutrient rich	LC3 puncta (FM) ↔	ATG5 ↓ ATG7 ↓ LC3-II ↑	p-AMPK ↑ p62 ↑	Inhibition of initiation	[128]
				starvation	LC3 puncta (FM) ↓	LC3-I and LC3-II ↑	p62 ↑↑	Inhibition of initiation	
**IIa**	HDAC4	siRNA	GC	nutrient rich	LC3 puncta (FM) ↑	BECN1 ↑ ATG7 ↑ LC3-II ↑	−3-MA: ATG7 ↑ +3-MA: ATG7 ↓ −3-MA: LC3-II ↑ +3-MA: LC3-II ↓	Induction. 48–72 h post-transfection	[82]
	HDACs 4 and 5	miRNA9 *	WM	nutrient rich	n.d.	RAB7 ↑ LC3B ↑	n.d.	Induced. 24 h post-transfection	[129]
	HDAC5	siRNA	mixed	nutrient rich	autophagic vesicles (EM) ↑ LC3 puncta (FM) ↑	LC3-II ↑	+NH4Cl: LC3-II ↑↑	Induced. 24–72 h post-transfection	[130]
**IIb**	HDAC6	siRNA	HeLa cells	starvation	LC3 puncta (FM) ↑	LC3-II ↑	p62 ↑ ac-LC3-II ↑	Blocked. 72 h post-transfection	[131]
	Hdac6	knockout	MEF	nutrient rich	autophagic vesicles (EM) ↑	LC3-II ↑	p62 ↑ mCherry-GFP-LC3 (FM): yellow ↑	Blocked—QC autophagy only	[132]
				starvation	n.d.	LC3-II ↑	mCherry-GFP-LC3 (FM): yellow ↔	Blocked—QC autophagy only	
				nutrient rich + proteasome inhibition	LC3 puncta (FM) ↑ LC3-ubiquitin overlapping puncta (FM) ↑	+MG132: LC3-II ↑↑	n.d.	Blocked—QC autophagy only	
	HDAC6	siRNA	breast cancer	nutrient rich	LC3 puncta (FM) ↑	LC3B ↔	n.d.	Blocked.	[134]
				nutrient rich + proteasome inhibition	+BORT: LC3 puncta (FM) ↓	+BORT: LC3B ↓	n.d.	Blocked.	
	Hdac6	knockout	mouse	nutrient rich + smoke	protein aggregates (FM) ↑	autophagosomes (EM) ↑ ^a^	+lysosomal protease inhibition: LC3B-II ↔	Blocked.	[135]
	Hdac6	knockout; siRNA	MEF	nutrient rich + mitochond. dysfunction	mitochondria (FM) ↑	n.d.	mitochondrial marker (Tom20;WB) ↑	Blocked. Impaired mitophagy	[136]
	HDAC10	siRNA	NB	nutrient rich	autophagic vesicles (EM) ↑ LC3 puncta (FM) ↑	LC3-II ↑ AO ↑ LAMP2 ↑	+CQ: LC3-II ↔ p62↑ EGFP-LC3 (FACS) ↓ mCherry-EGFP-LC3 (FM): yellow ↑	Blocked. 72–144 h post-transfection	[11]

^a^ Autophagosomes were quantified from EM images of ciliated cells. ImageJ software was used to calculate the number of autophagosomes per unit area (mm^2^).

Proposed models based on the current literature depicting the roles of different HDAC family members in autophagy as well as the steps in autophagy affected by inhibition of single or multiple HDAC isozymes. The initiation of autophagy is controlled by different signaling pathways, and mTOR is one of the key players, blocking autophagy in the setting of adequate nutrition and that is inhibited via AMPK or other signaling in times of stress or starvation. The ULK1/2, which are part of a multiprotein complex, are repressed by mTOR. Upon inhibition of mTOR, this complex induces autophagy. The initial nucleation phase, creating the phagophore, is controlled by a complex involving PI3KCIII and Beclin1—containing complexes. Following this, elongation of the phagophore membrane occurs through the coordination of multiple proteins, known as the ATG conjugation system. Then, LC3 I is conjugated to phosphatidylethanolamine. The lipidated form of LC3 I (LC3 II) is attached to both faces of the phagophore membrane. A complete double membrane autophagosome then fuses with the lysosome into the autophagolysosome, and the cargo-containing membrane compartment is lysed and degraded. **(A)** Class I and IIa HDACs are associated with inhibition of the initiation phase of autophagy. Through changes in transcription or expression of autophagy-related proteins important for nucleation, such as Beclin1, or elongation, such as ATG7 or LC3B, these family members inhibit autophagy initiation. Conversely, Class IIb HDACs have been found to promote the latter phase of autophagy, maturation. These family members have been associated with promoting the autophagosome delivery to, and subsequent fusion with lysosomes; **(B)** Class-I specific HDAC inhibitors impair the suppression of autophagy by their targets, thus promoting the initiation of autophagic flux. Meanwhile, pan-HDACi both promote initiation and block maturation simultaneously. Class IIb specific inhibitors block the latter phase of autophagy in context-dependent manners.

Abbreviations: mTOR, mammalian target of rapamycin; AMPK, adenosine monophosphate-activated protein kinase; ULK, uncoordinated 51-like kinase; ATG, autophagy-related; LC3 I, microtubule-associated protein 1 light chain 3; PI3KCIII, phosphoinositide 3 kinase class III; HDACs, histone deacetylases; HDACi, histone deacetylase inhibitors.

In the context of embryonal tumors of the nervous system, we found that HDAC6 knockdown did not increase the number of acidic vesicular organelles (AVOs) under nutrient-rich and proteasome-intact conditions [11]. However, knockdown of HDAC10 in neuroblastoma cells increased the accumulation of AVOs, led to accumulation of ROS, and impaired efficient fusion of autophagosomes with lysosomes under the same conditions [11]. HDAC10-depleted cells were additionally strongly sensitized to cytotoxic chemotherapy [11]. HDAC10 was found to interact physically with deacetylated Hsp70 family members, which are proteins important for lysosomal integrity during the stress response and delivery of proteins to be degraded [11]. The interaction resulted in deacetylation, and this mechanism may relate to the disruption of autophagic flux by HDAC10 knockdown and HDAC10 inhibition [11].

Overall, both HDAC6 and HDAC10 appear to be involved in the regulation of autophagy via deacetylase activity in the cytoplasm. Both HDACs seem to mainly interfere with autophagosome maturation and autophagic flux. For HDAC6, important functions in selective autophagy have been demonstrated, for HDAC10 the exact mechanism of autophagy regulation is still unresolved.

## 5. Targeting Autophagy with HDAC Inhibition in Cancer

In the previous section, the literature describing the role of HDAC isozymes in autophagy was reviewed. Now we turn to the literature examining the effects of HDAC inhibitors on autophagy in cancer models. A selection of recent papers is summarized in Table 3 and a summarizing model is presented in Figure 1B.

Many, though not all, of the authors using the broad-spectrum HDAC inhibitors conclude that autophagy is induced upon inhibitor treatment. However, the differing roles of the single HDAC isozymes described and the simultaneous inhibition of multiple isozymes by many of the compounds complicates prediction and interpretation of the results. In studies using the pan-HDACi vorinostat, autophagy was induced under nutrient-rich conditions in ovarian cancer cells [137], chondrosarcoma cells [138], cervical cancer cells [139], malignant peripheral nerve sheath tumor cells [140], as well as in the non-transformed cell line, murine embryonic fibroblasts [141]. One exception was a study examining the hematologic malignancy, Down syndrome acute megakaryocytic leukemia (DS-AMKL), where authors found that vorinostat as well as other pan-HDACi, panobinostat and TSA, inhibited autophagic flux [57]. In a colon cancer cell line, TSA was found to induce autophagy [42]. In two studies examining panobinostat treatment of breast cancer cells, autophagy was found to be induced, evidenced by increases in Beclin1 and LC3-II as well as a concomitant decrease in the autophagy substrate, p62 [142,143]. Similar results were found in a study examining colon cancer cells [144].

**Table 3:** Selected recent publications using HDACi in cancer treatment to augment autophagy. MEF—murine embryonic fibroblast; FM—fluorescent microscopy; mTOR—mammalian target of rapamycin; BAF—bafilomycin A1; chemo—chemotherapy: decitabine (DNA methyltransferase inhibitor); EM—electron microscopy; AO—acridine orange, acidotropic dye, stains late-stage autophagosomes; n.d.—not determined; 3-MA—3-methyladenine; LTR—LysotrackerRed, a membrane-permeable dye, highly selective for acidic organelles; DS-AMKL—Down syndrome-acute megakaryocytic leukemia; FACS—flow cytometric analysis; ROS—reactive oxygen species; Cyto-ID—a cationic amphiphilic dye selective for the autophagic compartment (autophagosomes plus autolysosomes); FACS—flow cytometric analysis; CQ—chloroquine; TSA—trichostatin A; m-RFP-GFP-LC3—in merged image: red fluorescence = autolysosomes, yellow fluorescence = autophagosome; DAPK—death-associated protein kinase; MPNST—malignant peripheral nerve sheath tumor; VPA—valproic acid; MDC—monodansylcadaverine: acidotropic dye, stains late-stage autophagosomes; MRT: malignant rhabdoid tumors; mCherry-EGFP-LC3—in merged image: red fluorescence = autolysosomes, yellow fluorescence = autophagosome; BORT—bortezomib.

Inhibitor	Target(s)	Conc. (µM)	Context	Stress Status	Methods for the Detection of Autophagy			Overall Effect on Autophagy	Citation
					Morphology	Quantification	Flux Studies		
vorinostat	pan	5–20	MEF	nutrient rich	LC3 puncta (FM) ↑	LC3-II ↑ LC3 mRNA ↑	mTOR blocked: p-p70S6K ↓ p62 ↓ +BAF: LC3-II ↑↑	Induced. 8–48 h treatment	[141]
vorinostat	pan	1–2	ovarian cancer	nutrient rich + chemo	vacuoles (EM) ↑ LC3 puncta (FM) ↑	AO ↑	n.d.	Induced. 24–120 h treatment	[137]
vorinostat	pan	2–50	chondro-sarcoma	nutrient rich	vacuoles (EM) ↑	LC3-II ↑	+3-MA: LC3-II ↓	Induced. 24–48 h treatment	[138]
vorinostat	pan	8	cervical cancer	nutrient rich	LTR (FM) ↑ LC3 puncta (FM) ↑	LC3-II ↑	n.d.	Induced. 24 h treatment	[139]
vorinostat	pan	1	DS-AMKL	nutrient rich	n.d.	ROS ↑ Cyto-ID (FACS) ↓	+CQ: LC3-GFP (FACS) ↓	Blocked.	[57]
TSA	pan	0.04–1	colon cancer	nutrient rich	n.d.	LC3-II ↑ ATG5 ↑ AO ↑	n.d.	Induced. 24 h treatment	[42]
				nutrient rich + radiation	n.d.	LC3-II ↑ AO ↑↑	n.d.	Induced. 24 h treatment	
TSA	pan	0.4	DS-AMKL	nutrient rich	n.d.	ROS ↑ LC3-GFP (FACS) ↑	n.d.	Blocked.	[57]
panobinostat	pan	0.02–0.05	triple negative breast cancer	nutrient rich	LC3 puncta (FM) ↑	BECN1 ↑ LC3-II ↑	p62 ↓ +CQ: p62 ↑	Induced. 16 h treatment	[143]
panobinostat	pan	0.1–0.4	DS-AMKL	nutrient rich	n.d.	Cyto-ID (FACS) ↓	+CQ: LC3-GFP (FACS) ↓	Blocked.	[57]
panobinostat	pan	0.1	breast cancer	nutrient rich	LC3 puncta (FM) ↑	BECN1 ↑ Vps34 ↑ LC3-II ↑	p62 ↓ m-RFP-GFP-LC3 (FM): red ↑	Induced. 24–48 h treatment	[142]
panobinostat	pan	0.05	colon cancer	nutrient rich	LC3 puncta (FM) ↑ AO (FM) ↑	LC3-II ↑	p62 ↓ +BAF: p62 ↑	Induced. 24–48 h treatment	[144]
				nutrient rich + DAPK	LC3 puncta (FM) ↑↑ AO (FM) ↑↑	LC3-II ↑↑	p62 ↓ +BAF: p62 ↑	Induced. 24–48 h treatment	
PCI-24781	pan	0.5	MPNST	nutrient rich	vacuoles (EM) ↑ AO (FM) ↑ LC3 puncta (FM) ↑	AO ↑ LC3-II ↑	+BAF: LC3-II ↑↑ +CQ: LC3-II ↑↑	Induced. 24 h treatment	[140]
VPA	Class I	2000	DS-AMKL	nutrient rich	n.d.	ROS ↑ ac-ATG7 ↑	12–17 h: LC3-GFP (FACS) ↓ 17–24 h: LC3-GFP (FACS) ↑	Induced early (12–17 h) Blocked later (17–48 h)	[57]
				starvation	n.d.	ROS ↔	LC3-GFP (FACS) ↔	No effect. 24 h treatment	
VPA	Class I	1000	glioma	nutrient rich	vacuoles (EM) ↑ LC3 puncta (FM) ↑ MDC puncta (FM)↑	LC3-II ↑ MDC ↑	+3-MA: LC3-GFP (FM) ↓ +3-MA: LC3-II ↓ +3-MA: MDC (FM) ↓	Induced. 48–96 h treatment	[145]
FK228	1, 2	0.148	cervical cancer	nutrient rich	vacuoles (EM) ↑ LC3 puncta (FM) ↑ MDC (FM) ↑ LTR (FM) ↑	LC3-II ↑	n.d.	Induced. 24 h treatment	[139]
FK228	1, 2	0.0025	MRT	nutrient rich	vacuoles (EM) ↑	LC3-II ↑	n.d.	Induced. 24–48 h treatment	[146]
Entinostat	1, 2, 3	3–5	colon cancer	nutrient rich	LC3 puncta (FM) ↑ vacuoles (EM) ↑	LC3-II ↑ ATG7 ↑ p-ERK ↑	n.d.	Induced. 2–24 h treatment	[147]
MGCD0103	1, 2, 3, 11	0.5 and 3	CLL	nutrient rich	n.d.	mRNA: ATG7 ↓ GABARAP ↓ WB: BECN1 ↓ ATG5 ↓ Cyto-ID (FACS) ↓	p-mTOR, early ↑ later ↓ Time-dependent: LC3-II ↓ p62 ↓ +CQ: LC3-II ↓ +CQ: p62 ↓	Inhibition of initiation. 2–48 h treatment	[148]
bufexamac	Class IIb	30	NB	nutrient rich	n.d.	AO ↑	mCherry-EGFP-LC3 (FM): yellow ↑	Blocked. 24 h treatment	[11]
bufexamac	Class IIb	30	MB	nutrient rich	n.d.	n.d.	p62 ↑ after 6 h mCherry-EGFP-LC3 (FM): yellow ↑	Blocked. 24 h treatment	Oehme, *unpublished data*
ST80	6	50	RMS	nutrient rich + proteasome inhibition	n.d.	+/− BORT: LTR (FACS) ↔	p62 ↑ +BORT: p62 ↑↑	Blocked—PQC. No change in flux. 48 h treatment	[149]
tubacin	6	2	cervical cancer	nutrient rich	LC3 puncta (FM) ↑	LC3-II ↑ ac-LC3-II ↑	p62 ↑ +CQ: LC3-II ↔	Blocked. 2–24 h treatment	[131]
				starvation	LC3 puncta (FM) ↑↑	ac-LC3-II ↑ (partial)	p62 ↑		

For inhibitors targeting class I isozymes as a collective, the outcome is often autophagy induction (Figure 1B). In yeast, inhibition of HDACs Rpd3 and Hda1, which are orthologues of Class I and II human HDACs, respectively, was found to induce autophagy, which in turn leads to degradation of DNA repair proteins [150]. In other studies examining hematologic malignancies [57] and also in normal cells, such as cardiomyocytes [151], HDAC inhibition suppressed autophagy. In the study examining HDACi in DS-AMKL, the authors found that VPA, which targets class I HDACs, inhibited autophagy under fed conditions, but had no effect when cells were starved [57]. In addition, atime-dependent effect was described, with HDAC inhibition resulting in induction at early time points and inhibition at later time points, which was also described by Xie and colleagues [152]. When glioma cells in a nutrient rich environment were treated with VPA, autophagy was induced at a later time point [145]. Similarly, the HDAC1 and 2 inhibitor, FK228, induced autophagy in cervical cancer and malignant rhabdoid tumor cells [139,146]. Entinostat, which inhibits HDACs 1, 2, and 3, induced autophagy in colon cancer cells [147]. In contrast, MGCD0103, which primarily inhibits HDAC1, but also HDACs 2, 3, and 11, inhibited autophagy via early mTOR activation and later degradation of autophagy-related proteins in a chronic lymphocytic leukemia model [148].

Intracellular targets are better defined for the class IIb isozymes and there are also more specific inhibitors for this class. Compounds inhibiting class IIb HDAC activity are associated with both inhibition and induction of autophagic flux. In HeLa cells, treatment with the HDAC6 inhibitor, tubacin, yielded increased acetylation of LC3-II, which was associated with decreased degradation of p62 and thus inhibition of autophagic flux [131]. Inhibition of HDAC6 with tubacin also led to a failure to degrade misfolded protein aggregates in murine neuroblastoma cells, very likely due to impaired retrograde transport of autophagic vacuoles and lysosomes, and LC3 recruitment to the autophagosomes membrane was impaired [153]. Using an HDAC6-specific inhibitor, ST80, resulted in autophagy inhibition in rhabdomyosarcoma cells [149]. In response to co-treatment with a proteasome inhibitor and ST80, some of the rhabdomyosarcoma cells upregulated BAG3 which induced autophagy and allowed the cells to resist succumbing to treatment [149]. Inhibiting only the catalytic activity of HDAC6 without disrupting the ubiquitin binding capacity of the protein led to a failure to inhibit trehalose-induced autophagy, which is mTOR independent and important for clearing protein aggregates, indicating an importance for the ubiquitin-binding domain in that context [154]. Likewise, in neuroblastoma cells, treatment with tubacin, which also specifically inhibits HDAC6 catalytic activity, did not result in an accumulation of AVOs, indicating that autophagic flux was not blocked under nutrient-rich and proteasome-active conditions [11]. Treatment with bufexamac, a class IIb inhibitor, led to accumulation of acidic vesicles and increased sensitivity to cytotoxic chemotherapeutic drugs in both neuroblastoma and medulloblastoma cells [11].

These results emphasize the importance of considering the regulation of autophagy. While in some cases the tumor is under metabolic stress, but in other cases, autophagy is upregulated even in the context of well-fed conditions (reviewed in [23]). Class I and IIa HDACs and their inhibitors appear to be involved in regulating the expression of proteins involved in autophagy via histone or transcription factor acetylation regulation, as well as potentially modulating the mechanistic target of rapamycin (mTOR) pathway. This is not completely clear, as some results are conflicting, and this may be a function not only of time points and different cell models, but also of inhibitor concentration employed. For instance, vorinostat was used in a broad range of concentrations, from 1–50 µM in the studies summarized in Table 3. Autophagy-inducing effects on the mTOR pathway were described in a study using a concentration at the high end of this range and one study showing that vorinostat inhibited autophagy used a low concentration. Class IIb HDACs and HDACi seem to have cytoplasmic roles in modulating autophagy, regulating the acetylation of key players such as HSP70 family members and LC3-II. The development of isozyme-specific HDAC inhibitors and a better understanding of the context-dependent effects of the individual HDAC isozymes on autophagic flux will provide more efficient options to kill cancer cells by exploiting this vulnerability in aspecifically-targeted manner.

## 6. Conclusion

Autophagy is a constitutive process that can become dysregulated in advanced malignant tumors. In some cases, active autophagy-mediated stress handling seems to provide the tumor cell with a mechanism for therapy resistance. Specific targeting of this therapy-hindering feature of autophagy represents an opportunity to hit an aggressive, highly metabolically active tumor cell and disrupt this therapy-interfering mechanism or even kill cells that obligatorily depend upon autophagy for survival.

Every member of the classical HDAC family has been associated with one or more cancer entities by virtue of changes in expression, primarily upregulation. The roles of these isozymes are diverse, and include effects on proliferation, differentiation, migration, as well as modulating response to oxidative and metabolic stress. Among these roles is the modulation of autophagy, including both induction and inhibition of autophagic flux.

Evidence points to the inhibition of class I HDACs leading to an induction of autophagy either via direct upregulation of autophagy-related genes, such as LC3 [141] and BECN1 [127], or indirectly via a stress-induced response. Inhibition of class IIb HDACs more likely inhibits autophagic flux at the level of autophagosome-autolysosome fusion via direct deacetylation of regulators of autophagy, such as ATG7, LC3, and heat shock proteins [11,57,87,131]. As class IIb selective inhibitors exist, targeting of these HDACs provides the opportunity to interfere with autophagy in aggressive tumors. Selective HDACi may be less toxic than pan-HDACi, as fewer cellular pathways are simultaneously targeted.

However, to our knowledge no study exists, which directly compares autophagic flux effects of selective class I HDACi with selective class II HDACi side-by-side under identical experimental conditions. This would further elucidate the nature of the different behavior of these compounds with respect to autophagic flux.

Pan-inhibitors that target at least both classes at the same time have been reported by some to induce autophagy and by others to inhibit it or even do both, in a time dependent-manner with induction followed by inhibition. The pan inhibitors target multiple isozymes, but the extent of inhibition of the individual isozymes varies with concentration, and at higher concentrations, stress-induction and off-target effects must also be considered. Additionally, it is important to bear in mind with the use of the pan-inhibitors that the simultaneous inhibition of multiple isozymes that are important in so many integral cellular processes favors autophagy induction via the resultant stress experienced by the cell. With class I and pan-HDACi being associated with autophagy induction, among other anti-tumor effects including apoptosis, cell-cycle arrest, and differentiation, the combination of these agents with inhibitors of autophagic flux, such as the lysosomal inhibitor HCQ, holds much promise. One such combination, vorinostat plus HCQ, was tested and some patients did benefit in an early phase clinical trial [155].

The challenge still remains to reliably identify tumors that are addicted to autophagy. Those tumors that are autophagy-addicted will be more likely to respond to therapy targeting this process. Future studies that continue to elucidate how and in which tumor entities individual HDAC family members modulate autophagic flux will be important for identifying the contexts in which implementing specific inhibitors will be most effective. Specifically-targeted inhibition is desirable not only to reduce unwanted off-target effects within the cell, but also to minimize adverse events experienced by the patient.
